# Physiological and psychological determinants of whole-body endurance exercise following short-term sustained operations with partial sleep deprivation

**DOI:** 10.1007/s00421-018-3869-0

**Published:** 2018-04-23

**Authors:** Michail E. Keramidas, Magnus Gadefors, Lars-Ove Nilsson, Ola Eiken

**Affiliations:** 1 Department of Environmental Physiology, Swedish Aerospace Physiology Center, Royal Institute of Technology-KTH, Berzelius väg 13, 171 65 Solna, Sweden; 2 Military Academy Karlberg, Stockholm, Sweden

**Keywords:** Autonomic dysfunction, Cerebral oxygenation, Effort, Fatigue, Motivation, Muscle oxygenation

## Abstract

**Purpose:**

The study examined the effects of short-term field-based military training with partial sleep deprivation on whole-body endurance performance in well-trained individuals.

**Methods:**

Before and after a 2-day sustained operations (SUSOPS), 14 cadets performed a 15-min constant-load cycling at 65% of peak power output (PPO; CLT_65_), followed by an exhaustive constant-load trial at 85% of PPO (CLT_85_). Physiological [oxygen uptake ($\dot {V}$O_2_), heart rate (HR), mean arterial pressure (MAP), cardiac output (CO), and regional oxygenation (TOI) in the frontal cerebral cortex and vastus lateralis muscle] and psychological [effort perception (RPE), affective valence (FS), and perceived activation (FAS)] variables were monitored during exercise.

**Results:**

SUSOPS reduced time to exhaustion in CLT_85_ by 29.1% (*p* = 0.01). During the CLT_65_ trial, SUSOPS potentiated the exercise-induced elevations in $\dot {V}$O_2_ and HR (*p* < 0.05), and blunted MAP (*p* = 0.001). CO did not differ between trials. Yet, towards the end of both CLT_85_ trials, CO tended to decline (*p* ≤ 0.08); a response that occurred at an earlier stage in the SUSOPS trial. During CLT_65_, SUSOPS altered neither cerebral nor muscle TOI. The SUSOPS CLT_85_ trial, however, was terminated at similar leg-muscle deoxygenation (*p* > 0.05) and lower prefrontal cortex deoxygenation (*p* < 0.01). SUSOPS increased RPE at submaximal intensities (*p* = 0.05), and suppressed FAS and FS throughout (*p* < 0.01).

**Conclusions:**

The present findings indicate, therefore, that a brief period of military sustained operations with partial sleep deprivation augment cardiorespiratory and psychological strain, limiting high-intensity endurance capacity.

## Introduction

Military and emergency-response personnel are often required to perform sustained and demanding work in environmental extremes, while provisions for full recovery are limited. During such multi-day tasks, individuals may be exposed to several behavioural stressors, including physical and mental exertion, partial or total sleep deprivation, and caloric deficit (i.e., energy intake is lower than expenditure), which, independently or interactively, might result in functional impairments (for reviews, see Henning et al. 2011; Vrijkotte et al. 2016; Montain and Young 2003). Specifically, military-based studies have suggested that a prolonged period of sustained operations (SUSOPS) degrades cognitive and physical performance; thus, aerobic work capacity is typically suppressed (Guezennec et al. 1994; Nindl et al. 2002). Physiological and psychological modifications, such as low energy substrate availability (Smith et al. 2016; Rognum et al. 1981), muscle-mass loss (Johnson et al. 1994), hypovolemia and/or hypohydration (Lieberman et al. 2005; Wittels et al. 1996), functional peripheral deteriorations (e.g., impaired mitochondrial efficiency; Fernstrom et al. 2007), and decreased motivation and enhanced effort perception (Lucas et al. 2009; Lieberman et al. 2005, 2006), have been regarded as potential determinants of physical performance in such multi-stressor conditions.

The SUSOPS effect on endurance capacity seems to be dictated primarily by the severity of energy and sleep deprivation encountered. For instance, short-term (≤ 10 days) periods of low-to-moderate hypocaloria cause minimal, if at all, change in aerobic capacity (Dohm et al. 1986; Knapik et al. 1987; Guezennec et al. 1994). Friedl (1995) has argued that body mass losses of at least 5–10% might be required to adversely affect performance. Moreover, although it is well established that partial sleep deprivation deteriorates cognitive and mental performance, its impact on endurance capacity is equivocal; a few studies have observed an impairment, while others have shown no change (for review, see Fullagar et al. 2015). Hence, information is scarce regarding effects of a short-term SUSOPS, during which the intensities of the stressors are moderate, on aerobic capacity.

The purpose of the present study, therefore, was to determine whether, and to what extent, a brief period of multi-stressor military training would influence high-intensity, whole-body, endurance exercise in well-trained individuals. For this purpose, central and peripheral haemodynamics, and perceptual and affective reactions were monitored during exhaustive constant-load cycle ergometry before and immediately after a 2-day SUSOPS with partial sleep deprivation. We hypothesized that, despite its short duration and relatively moderate intensity, SUSOPS would increase cardiorespiratory and psychological strain, thereby precipitating a reduction in maximal exercise tolerance.

## Methods

### Ethics approval

The experimental protocol was approved by the Human Ethics Committee of Stockholm (2017/1:8), and conformed to the standards set by the Declaration of Helsinki. The study was part of the course “Applied Physical Training Theory for Warfare” of the school program of the Military Academy Karlberg (Sweden). Subjects were informed in detail about the experimental procedures, and gave their consent.

### Subjects

Fourteen healthy cadets of the Swedish Armed Forces [13 males and 1 female; mean (standard deviation; SD) age 25 (2) years, stature 182.1 (7.6) cm, body mass 79.4 (9.5) kg, body mass index 23.9 (2.0) kg m^−2^, body fat 10.0 (3.2)%] volunteered to participate in the study. They were non-smokers, and free of any cardiorespiratory, metabolic, or neurological disease.

### Experimental protocol

All experimental trials were performed in a laboratory of the Department of Environmental Physiology, Royal Institute of Technology (Solna, Sweden). Four days prior to the main exercise trials, subjects were thoroughly familiarized with the equipment and experimental procedure; anthropometry measurements and an incremental exercise trial to exhaustion were also performed (see below for details). Two days before (CON) and within 1–6 h after the end of 2-day SUSOPS, subjects performed two constant-load exercise trials (see below). Subjects were instructed to maintain their normal sleep/wake and activity patterns before the CON trial. All the exercise trials were performed on an electrically braked cycle ergometer (Daum Electronic GmbH, Furth, Germany). The environmental conditions in the laboratory were kept constant: the mean temperature, relative humidity, and barometric pressure were 21.0 (0.7) °C, 25 (6)%, and 753 (11) mmHg, respectively.

The SUSOPS took place at Berga (Muskö Naval Base, Sweden) during the first week of March. It was neither raining nor snowing; the mean ambient temperature, relative humidity, and barometric pressure were 2.2 (1.7) °C, 86 (8)%, and 748 (6) mmHg, respectively. SUSOPS commenced at 06:30 AM and finished 51 h later at 08:00 AM. During this period, subjects conducted almost continuous military-relevant field tasks in varying terrain, requiring moderate physical and mental effort. Subjects were only allowed to take short naps; the total sleeping time was estimated to be ~ 5 h (in day 1: from 16:00 to 18:00 PM, in day 2: from 03:00 to 05:00 AM and from 21:00 to 21:40 PM). Food was limited to 3 meals day^−1^, during which subjects were allowed to drink coffee; they were instructed, however, to refrain from consuming caffeine and eating for a minimum of 4 h prior to the exercise trials. Subjects consumed ~ 3600 kcal day^−1^, consisting of ~ 60% carbohydrates, ~ 20% fat and ~ 20% proteins, and were allowed to drink water ad libitum. Although no measures of energy expenditure were performed in the field, based on reports from the previous SUSOPS studies, in which the recorded values of energy expenditure typically ranged between 4000 and 8000 kcal day^−1^ (Castellani et al. 2006; Tassone and Baker 2017; Tharion et al. 2005), it is reasonable to assume that, in the present SUSOPS, subjects were in energy deficit, at least to some degree.

#### Anthropometrics

All measurements were performed during the preliminary visit. In addition, body mass was monitored before each experimental trial using an electronic scale with an accuracy 0.01 kg (Vetek, Väddö, Sweden). Height was measured with a stadiometer. Skinfold thicknesses were measured with a skinfold caliper (Harpenden, UK) at seven right-side locations: triceps, subscapular, chest, suprailiac, abdominal, front thigh, and midaxillary. Percent body fat was calculated according to the equation of Jackson and Pollock (1978).

#### Incremental-load trial

The trial commenced with a 2-min rest period, followed by a 2-min warm-up at a workload of 60 W. Thereafter, the load was increased by 25 W min^−1^ until exhaustion. Attainment of peak oxygen uptake ($\dot {V}$O_2peak_), defined as the highest $\dot {V}$O_2_ averaged over 30 s, was confirmed according to the following criteria: (1) severe fatigue or exhaustion resulting in an inability to maintain exercise at a given work rate (cycling cadence < 60 rpm) and (2) a subjective rating of effort perception at or near maximal. Peak power output (PPO) was calculated by the equation: PPO = PO_FINAL_ + (*t*/60 × 25 W), where PO_FINAL_ is the last workload completed, and *t* is the number of seconds for which the final, uncompleted workload was sustained.

#### Constant-load trials

The trial began with a 2-min rest period on the ergometer to record baseline values. Thereafter, subjects were asked to complete a 2-min warm-up at an individualized work rate of 1 W kg^−1^ body weight [mean power output = 79 (10) W]. Subsequently, they performed a 15-min constant-load exercise bout at an intensity of 65% of their PPO [CLT_65_; mean power output = 231 (25) W], which was immediately followed by an exhaustive constant-load bout at 85% of PPO [CLT_85_; mean power output = 302 (33) W]. For each subject, all trials were performed at the same absolute intensity. During CLT_65_, subjects pedaled at a cadence between 65 and 70 rpm, whereas during CLT_85_, they selected their preferred pedal cadence (between 60 and 90 rpm). The investigator terminated the trial when the pedal cadence dropped below 70% of the self-selected cadence for ≥ 5 s. During all trials, subjects received verbal encouragement always in the same manner and by the same investigator. The height of the cycle seat was maintained constant for each subject, who always exercised seated on the cycle ergometer to minimize changes in muscle recruitment.

### Physiological measurements

#### Respiratory variables

During the exercise trials, subjects were equipped with a facemask to enable monitoring of respiratory gas continuously. $\dot {V}$O_2_, carbon dioxide production ($\dot {V}$CO_2_), respiratory exchange ratio (RER), expired ventilation ($\dot {V}$E), ventilatory equivalent for oxygen ($\dot {V}$E/$\dot {V}$O_2_), ventilatory equivalent for carbon dioxide ($\dot {V}$E/$\dot {V}$CO_2_), tidal volume (*V*_T_), respiratory frequency (*f*_R_), and partial pressure of end-tidal carbon dioxide (*P*_ET_CO_2_) were measured online using a metabolic unit (Quark PFT; Cosmed, Rome, Italy). The gas analysers and pneumotachograph were calibrated before each trial with two different gas mixtures and a 3-L syringe, respectively.

#### Heart rate (HR), cardiac output (CO), and stroke volume (SV)

HR was measured using 3-lead electrocardiography, and CO was determined using an electrical impedance cardiography system (Physioflow® PF05 Lab1™, Manatec Biomedical, Paris, France). The method measures changes in thoracic impedance during cardiac ejection to calculate SV. Six electrodes were placed at the base of the neck and on the chest wall, always by the same investigator. The calibration procedure was carried out before each trial, while subjects rested on the cycle ergometer.

#### Near-infrared spectroscopy (NIRS)

Cerebral and leg-muscle oxygenation were monitored with a three-wavelength (735, 810, and 850 nm) NIRS device (NIRO-200NX, Hamamatsu Photonics, Japan). The cerebral probe was positioned over the left prefrontal cortex between the first frontal polar and the third frontal locations, as determined using the modified international 10–20 system for electroencephalograms. The leg-muscle probe was placed above the vastus lateralis muscle, 15 cm above the proximal line of the patella and 5 cm lateral to the midline of the right thigh. The NIRS probes were always positioned by the same investigator. The probes consisted of one emitter and one detector housed in a black, plastic holder that was stabilised on the shaved and cleaned skin with double-sided adhesive tape. A bandage covered and stabilised each probe holder to reduce the intrusion of external light and the loss of transmitted NIR light from the measuring area. The interoptode distance was kept at 4 cm to minimize the influence of skin blood flow (Hampson and Piantadosi 1988). Skinfold thickness [mean (SD) 12.8 (5.2) mm] was measured between the NIRS optodes at the locomotor site using a caliper (Harpenden, UK). The theory, limitations, and reliability of the NIRS system during exercise have been described previously (Boushel et al. 2001). The modified Beer–Lambert law was used to determine concentration changes in oxyhaemoglobin (Δ[HbO_2_]) and deoxyhaemoglobin (Δ[HHb]). Based on the NIR spatially resolved spectroscopy, the tissue oxygen index (TOI) was also calculated. All the NIRS data were recorded continuously at 5 Hz, and expressed relative to the resting period of each trial.

#### Arterial pressures

Beat-to-beat systolic (SAP), diastolic (DAP), and mean (MAP) arterial pressures were obtained by finger photoplethysmography (Finometer, Finapres Medical Systems BV, Amsterdam, The Netherlands) during the 2-min rest period and until the 12th minute of CLT_65_. The finger cuff was placed on the middle phalanx of the middle finger of the right hand, which was kept on a hand support to avoid compression against the handlebars. The reference pressure transducer was positioned at the level of the heart. A brachial cuff was attached on the same arm, and the calibration process was performed according to the manufacturer’s instructions before each trial.

#### Blood lactate concentration ([La])

At the last minute of CLT_65_ and 2 min after the termination of CLT_85_, capillary blood was sampled from the index fingertip to measure [La]. The skin was punctured with a lancet (Accu-Check, Scoftclix Pro, Basel, Switzerland), the second drop of blood was placed on a strip (BM-lactate, Roche, Basel, Switzerland) and immediately analyzed with a portable analyser (Accutrend Lactate, Roche, Basel, Switzerland).

### Psychological measurements

#### Before and after exercise

Ten minutes before and ~ 5 min after the end of CLT_85_, subjects were requested to fill out the following questionnaires, based on how they felt at that particular moment: (1) the Profile of Mood States-Short Form (POMS-SF; Shacham 1983), which is a 37-item self-evaluation questionnaire of six subscales; tension–anxiety, depression–dejection, anger–hostility, vigor–activity, fatigue–inertia, and confusion–bewilderment. The description of subjects’ feelings was provided based on a five-point scale with anchors from 0—“not at all” to 4—“extremely”. (2) The Multidimensional Fatigue Inventory (MFI; Smets et al. 1995), which is a 20-item self-rating multidimensional inventory measuring different aspects of fatigue: general fatigue, physical fatigue, reduced activity, reduced motivation, and mental fatigue. Each subscale contains four items and the answer ranges from 1—“yes, that is true” to 5—“no, that is not true”. Before the exercise trials, subjects were also asked to rate their perception of sleepiness on the Stanford Sleepiness Scale (Hoddes et al. 1973). All the questionnaires were presented in a hardcopy format, and were explained to the subjects by the same investigator prior to each trial. Subjects replied to the questions in 5–7 min, while seated comfortably in a chair and at a distance from the investigators.

#### Before and during exercise

During the 2-min rest period on the cycle ergometer, at 2-min intervals during CLT_65_, and at 1-min interval during CLT_85_, subjects provided ratings for perceived exertion (RPE) using the 11-point scale ranging from 0—“nothing at all” to 10—“maximum”. At the same time intervals, the acute affective responses were also monitored by means of: (1) the feeling scale (FS; Hardy and Rejeski 1989), which is an 11-point scale ranging from −5—“very bad” to + 5—“very good”, and measures affective valence along a pleasure–displeasure continuum and (2) the felt arousal scale (FAS; Svebak and Murgatroyd 1985), which measures perceived activation along a 6-point scale ranging from 1—“low arousal” to 6—“high arousal”.

### Statistical analyses

For technical reasons, the NIRS data from thirteen subjects were analyzed and presented in the current report. Considering the inter- and intra-individual variability in the duration of CLT_85_, data were expressed as functions of mean absolute exercise time, and relative to the duration of the SUSOPS CLT_85_ trial. Statistical analyses were performed using Statistica 8.0 (StatSoft, Tulsa, OK, USA). All data were tested for normal distribution with the Kolmogorov–Smirnov test. A two-way (trial × time) general linear model repeated measures ANOVA was used to examine differences in all physiological and psychological variables. Mauchly’s test was conducted to assess the sphericity, and the Greenhouse–Geisser *ε* correction was used to adjust the degrees of freedom when the assumption of sphericity was not satisfied. When ANOVA revealed a significant interaction or main effect, pairwise comparisons were performed with Dunnett post hoc test. A paired sample Student’s *t* test was used to detect changes in the duration of CLT_85_. All data are presented as mean (SD). In addition, where appropriate, the 95% confidence interval (95% CI) of the difference was included. The *α*-level of significance was set a priori at 0.05.

## Results

### Incremental-load trial

The average values of $\dot {V}$O_2peak_ and PPO were 50.7 (5.6) mL kg^−1^ min^−1^ and 355 (39) W, respectively. The average peak values of HR, $\dot {V}$E, and RPE were 186 (9) beats min^−1^, 165.6 (29.7) L min^−1^ and 10 (0), respectively.

### Constant-load trials

All subjects completed the 2-day SUSOPS, which reduced body mass by ~ 1% [CON = 79.7 (9.4) kg, SUSOPS = 78.9 (9.5) kg; *p* = 0.003], and increased perceived sleepiness [CON = 2.1 (0.9), SUSOPS = 5.0 (1.0); *p* < 0.001].

**Fig. 1:**
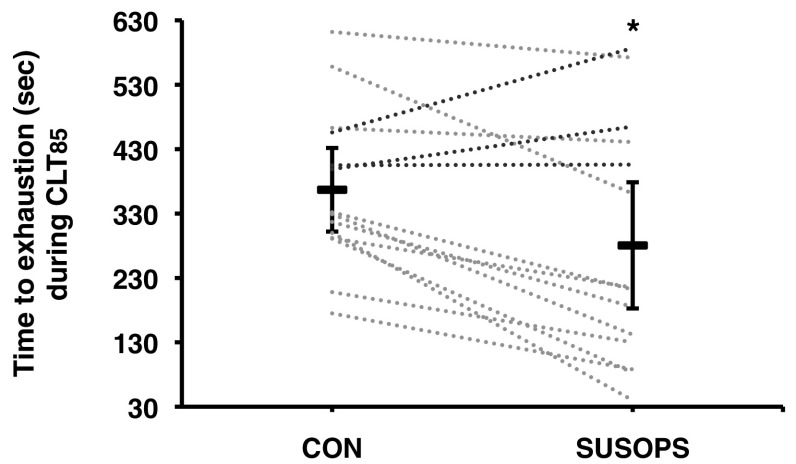
Mean (95% confidence intervals; black line) and individual (shaded dotted lines) time to exhaustion during a constant-load trial at 85% of peak power output (CLT_85_) performed before (CON) and after a 2-day sustained operations (SUSOPS) with partial sleep deprivation. The red lines indicate the three “outliers”, in whom the exercise performance was enhanced after SUSOPS. *Significantly different from CON trial (*p* = 0.01)

All subjects completed the CLT_65_ trial. SUSOPS reduced time to exhaustion in the CLT_85_ trial by 29.1% (95% CI: − 11.9, − 46.3%; *p* = 0.01); time to exhaustion was shorter in 11 out of 14 subjects (Fig. 1). Mean cycling cadence was lower (*p* = 0.04) in the SUSOPS [73 (8) rpm] than in CON [77 (8) rpm] CLT_85_ trial.

#### Physiological responses

**Fig. 2:**
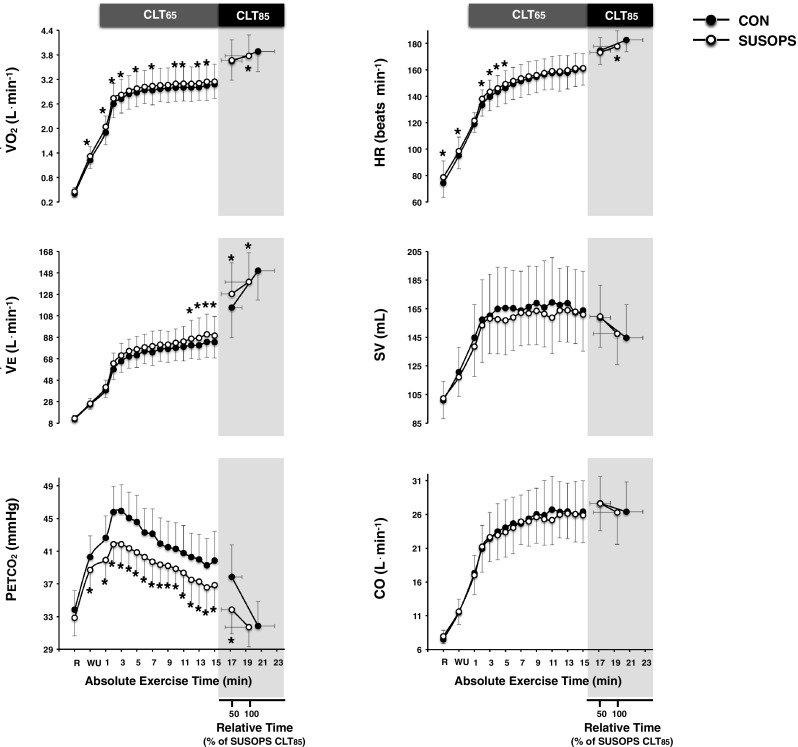
Mean (SD) values of oxygen uptake ($\dot {V}$O_2_), minute ventilation ($\dot {V}$E), partial pressure of end-tidal carbon dioxide (*P*_ET_CO_2_), heart rate (HR), stroke volume (SV), and cardiac output (CO) obtained during the 15-min constant-load trial at 65% (CLT_65_) and the exhaustive constant-load trial at 85% (CLT_85_) of peak power output performed before (CON) and after a 2-day sustained operations (SUSOPS) with partial sleep deprivation. Data are expressed as functions of mean absolute exercise time, and relative to the duration of the SUSOPS CLT_85_ trial. Data in all conditions were significantly different over time. *Significantly different from CON trial (*p* ≤ 0.05)

Cardiorespiratory variables are summarised in Table 1 and Fig. 2. SUSOPS increased the rates of the exercise-induced elevations in $\dot {V}$O_2_ (*p* = 0.03) and $\dot {V}$E (*p* = 0.01). However, at the point of exhaustion, $\dot {V}$O_2_, $\dot {V}$E, $\dot {V}$CO_2,_ and *V*_T_ were lower in the SUSOPS trial (*p* ≤ 0.01). At submaximal intensities, *P*_ET_CO_2_ was diminished by SUSOPS (*p* < 0.001), whereas the peak values of *P*_ET_CO_2_ did not differ between trials. RER was reduced by SUSOPS throughout (*p* < 0.001).

**Table 1:** Mean (SD) values of oxygen uptake ($\dot {V}$O_2_), carbon dioxide production ($\dot {V}$CO_2_), respiratory exchange ratio (RER), ventilatory equivalents for oxygen ($\dot {V}$E/$\dot {V}$O_2_) and carbon dioxide ($\dot {V}$E/$\dot {V}$CO_2_), respiratory frequency (*f*_R_), tidal volume (*V*_T_) and lactate concentration ([La]) obtained during the 15-min constant-load trial at 65% (CLT_65_) and the exhaustive constant-load trial at 85% (CLT_85_) of peak power output performed before (CON) and after a 2-day sustained operations (SUSOPS) with partial sleep deprivation

	CON trial			SUSOPS trial		
	Rest	CLT65	Peak at CLT85	Rest	CLT65	Peak at CLT85
$\dot {V}$O_2_ (mL min^−1^ kg^−1^)	5.1 (1.0)	36.0 (3.3)	48.9 (4.4)	5.7 (0.8)	37.5 (3.8)*	48.1 (6.0)
$\dot {V}$CO_2_ (L min^−1^)	0.35 (0.08)	2.74 (0.33)	4.03 (0.49)	0.36 (0.08)	2.71 (0.36)	3.74 (0.50)*
RER	0.88 (0.06)	0.96 (0.04)	1.04 (0.05)	0.79 (0.07)*	0.92 (0.02)*	0.99 (0.06)*
$\dot {V}$E/$\dot {V}$O_2_	29.2 (2.6)	25.3 (2.2)	38.5 (3.9)	27.6 (3.8)	26.2 (2.1)	36.9 (4.0)
$\dot {V}$E/$\dot {V}$CO_2_	33.3 (2.3)	26.4 (1.8)	37.1 (4.1)	34.8 (3.1)	28.6 (2.1)*	37.2 (3.8)
*f*_R_ (breaths min^−1^)	14 (3)	28 (3)	56 (9)	14 (3)	31 (5)	57 (11)
*V*_T_ (L)	0.9 (0.3)	2.6 (0.4)	2.7 (0.4)	0.9 (0.2)	2.5 (0.4)	2.5 (0.4)*
[La] (mmol L^−1^)	–	8.0 (2.4)	11.7 (2.4)	–	6.0 (1.8)*	8.8 (2.0)*

*Significantly different from CON trial (*p* ≤ 0.05)

**Fig. 3:**
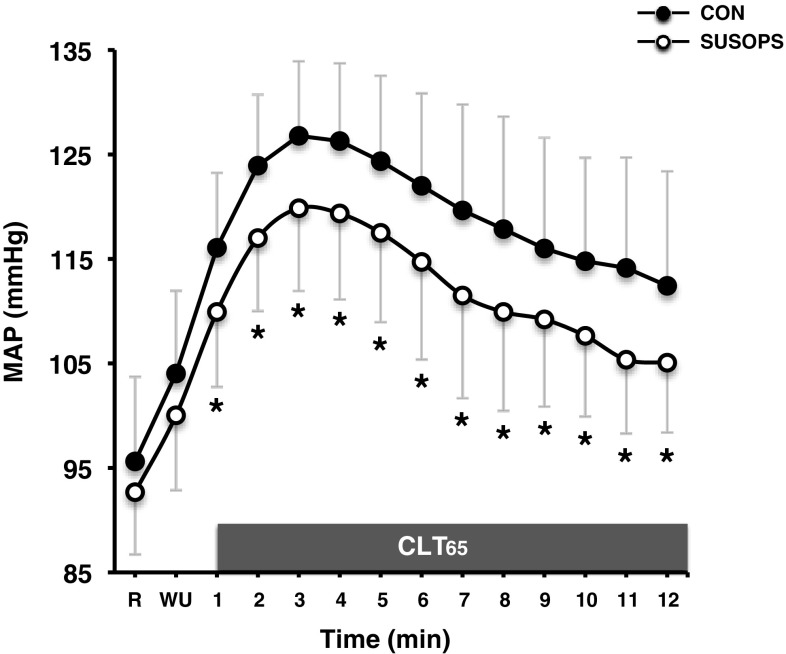
Mean (SD) values of mean arterial pressure (MAP) obtained until the 12th minute of the constant-load trial at 65% of peak power output (CLT_65_) performed before (CON) and after a 2-day sustained operations (SUSOPS) with partial sleep deprivation. Data in all conditions were significantly different over time. *Significantly different from CON trial (*p* = 0.001)

At rest and during the initial portion (up to the 4th minute) of CLT_65_, HR was augmented by SUSOPS (*p* < 0.01). The SUSOPS CLT_85_ trial, however, was terminated at lower peak HR (*p* < 0.001). [La] was reduced by SUSOPS (*p* < 0.001). SUSOPS altered neither SV (*p* = 0.46) nor CO (*p* = 0.68). At the end of both CLT_85_ trials, SV dropped [CON = − 14.1 (95% CI: − 6.8, − 21.4) mL; *p* = 0.003, SUSOPS = − 12.0 (95% CI: − 4.4, − 19.6) mL; *p* = 0.02], and CO tended to decline [CON = −1.27 (95% CI: 0.06, − 2.59) L min^−1^; *p* = 0.08, SUSOPS = − 1.33 (95% CI: − 0.06, − 2.59) L min^−1^; *p* = 0.06]. The resting values of arterial pressures did not differ between trials. However, SUSOPS blunted the exercise-mediated elevation in SAP [CON = 176 (13) mmHg, SUSOPS = 166 (12) mmHg; *p* = 0.05], DAP [CON = 88 (6) mmHg, SUSOPS = 81 (6) mmHg; *p* < 0.01] and MAP (*p* = 0.001; Fig. 3).

**Fig. 4:**
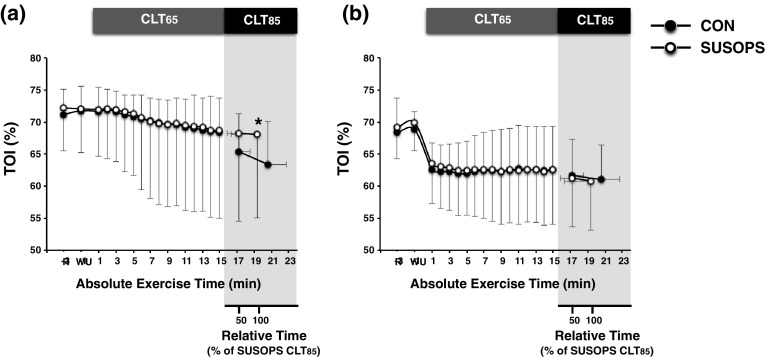
Mean (SD) values of tissue oxygen index (TOI) of cerebral frontal cortex (**a**) and vastus lateralis muscle (**b**) obtained during the 15-min constant-load trial at 65% (CLT_65_) and the exhaustive constant-load trial at 85% (CLT_85_) of peak power output performed before (CON) and after a 2-day sustained operations (SUSOPS) with partial sleep deprivation. Data are expressed as functions of mean absolute exercise time, and relative to the duration of the SUSOPS CLT_85_ trial. Data in all conditions were significantly different over time. *Significantly different from CON trial (*p* ≤ 0.05) (*n* = 13)

During CLT_65_, cerebral TOI did not differ between trials; yet, at the end of the trial, the TOI drop was attenuated by SUSOPS (*p* = 0.006; Fig. 4a). During CLT_65_, cerebral Δ[ΗbO_2_] [CON = 10.7 (4.5) µM, SUSOPS = 10.2 (4.9) µM; *p* = 0.60] and Δ[ΗHb] [CON = 0.6 (1.3) µM, SUSOPS = 0.9 (1.3) µM; *p* = 0.67] did not vary between trials. At the point of exhaustion, the exercise-induced elevation in cerebral Δ[HbO_2_] was blunted by SUSOPS [CON = 21.9 (6.2) µM, SUSOPS = 18.4 (7.6) µM; *p* < 0.01]. Muscle TOI (Fig. 4b), Δ[ΗbO_2_] [CLT_65_ : CON = − 0.6 (4.4) µM, SUSOPS = − 1.8 (3.7) µM; peak at CLT_85_ : CON = 0.3 (6.2) µM, SUSOPS = − 0.8 (4.4) µM], Δ[ΗHb] [CLT_65_ : CON = 3.6 (6.0) µM, SUSOPS = 2.4 (4.5) µM; peak at CLT_85_ : CON = 5.6 (6.8) µM, SUSOPS = 5.1 (5.2) µM] values were not affected by SUSOPS (*p* > 0.05).

#### Perceptual responses

The mean values of the POMS-SF subscales are presented in Table 2. SUSOPS raised confusion and fatigue, and impaired vigor (*p* ≤ 0.001). SUSOPS also augmented depression and anger (*p* ≤ 0.01), which, however, were ameliorated by the constant-load trials. The perceived tension remained unaltered by SUSOPS.

**Table 2:** Mean (SD) values of the Profile of Mood States-Short Form (POMS-SF) subscales pre and post constant-load exercise trials performed before (CON) and after a 2-day sustained operations (SUSOPS) with partial sleep deprivation

	CON trial		SUSOPS trial	
	Pre-exercise	Post-exercise	Pre-exercise	Post-exercise
Tension	4.3 (3.3)	1.6 (1.5)^†^	4.1 (3.2)	2.5 (2.2)
Depression	1.3 (3.2)	1.1 (2.7)	3.3 (4.1)*	2.0 (2.3)^†^
Vigor	13.1 (5.1)	13.0 (4.9)	5.9 (3.1)*	6.9 (5.0)*
Anger	0.2 (0.4)	0.4 (0.9)	2.4 (2.9)*	1.0 (1.5)^†^
Confusion	1.0 (1.1)	1.5 (2.1)	4.9 (4.3)*	5.0 (5.4)*
Fatigue	2.9 (3.6)	9.9 (2.3)^†^	11.7 (4.0)*	14.2 (4.2)*

^†^Significantly different from pre-exercise measures (*p* ≤ 0.01)

*Significantly different from CON trial (*p* ≤ 0.01)

The mean values of MFI subscales are summarised in Table 3. General, physical, and mental fatigue, and reduced activation and motivation were exacerbated by SUSOPS throughout (*p* ≤ 0.01).

**Table 3:** Mean (SD) values of the Multidimensional Fatigue Inventory (MFI) subscales pre and post constant-load exercise trials performed before (CON) and after a 2-day sustained operations (SUSOPS) with partial sleep deprivation

	CON trial		SUSOPS trial	
	Pre-exercise	Post-exercise	Pre-exercise	Post-exercise
General fatigue	8.5 (3.4)	10.0 (3.2)	16.1 (2.1)*	14.5 (2.8)*
Physical fatigue	7.9 (3.4)	8.2 (2.7)	12.8 (5.0)*	12.5 (4.5)*
Reduced activation	8.6 (3.1)	8.5 (3.0)	11.4 (3.4)*	11.4 (3.1)*
Reduced motivation	8.9 (3.1)	8.4 (2.4)	13.2 (3.4)*	13.8 (3.8)*
Mental fatigue	8.0 (2.7)	8.5 (2.4)	12.4 (3.5)*	13.0 (4.2)*

*Significantly different from CON (*p* ≤ 0.01)

**Fig. 5:**
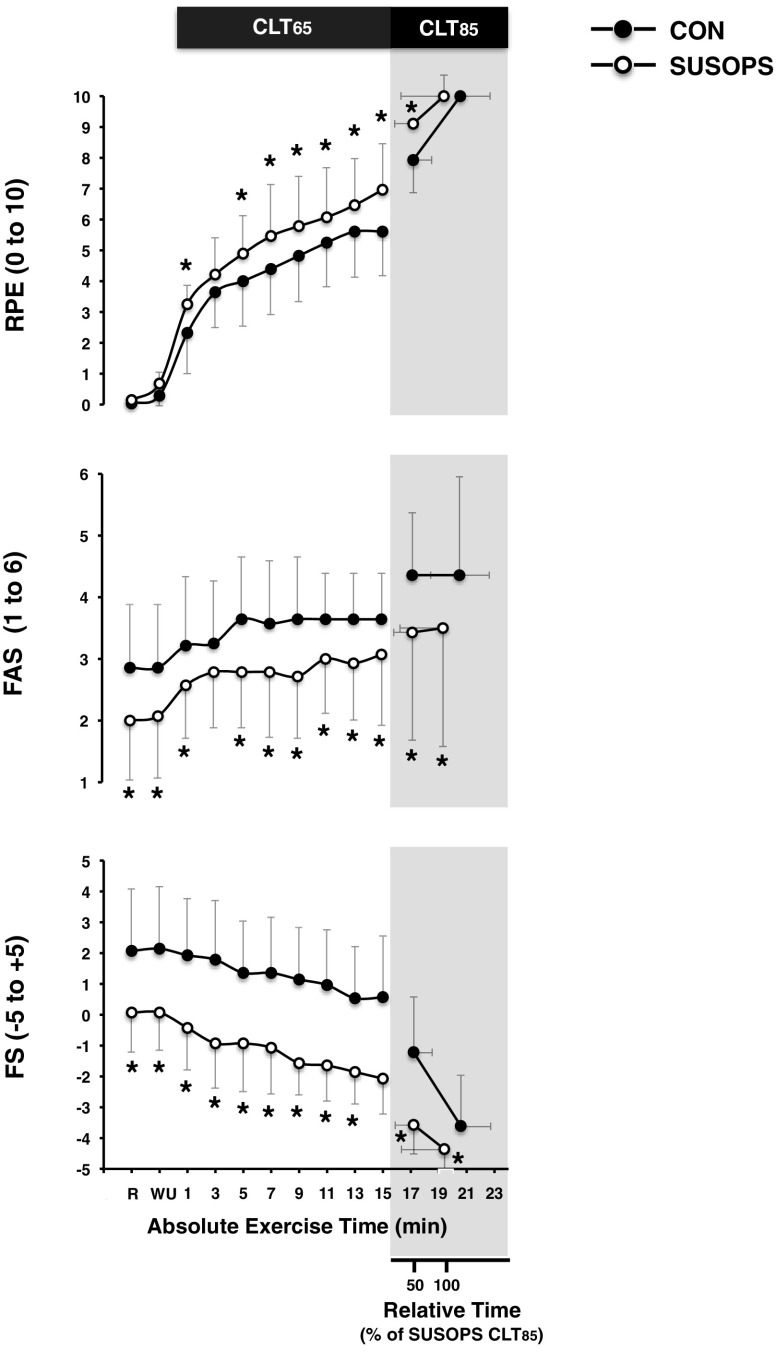
Mean (SD) ratings of perceived exertion (RPE), arousal (FAS) and affective valence (FS) obtained during the 15-min constant-load trial at 65% (CLT_65_) and the exhaustive constant-load trial at 85% (CLT_85_) of peak power output performed before (CON) and after a 2-day sustained operations (SUSOPS) with partial sleep deprivation. Data are expressed as functions of mean absolute exercise time, and relative to the duration of the SUSOPS CLT_85_ trial. Data in all conditions were significantly different over time. *Significantly different from CON trial (*p* ≤ 0.05)

SUSOPS increased the rate of the exercise-mediated elevation in RPE (*p* = 0.05); yet, at the point of exhaustion, RPE was identical in the two trials (Fig. 5). FAS and FS were suppressed throughout the SUSOPS trials (*p* < 0.01; Fig. 5).

## Discussion

The main finding of the study was that a 2-day field-based military training with partial sleep deprivation increased cardiorespiratory and psychological strain, and reduced the high-intensity constant-load cycling capacity in well-trained individuals. In line with the previous SUSOPS studies (Nindl et al. 2002; Lieberman et al. 2006; Guezennec et al. 1994), the present results demonstrated that a relatively brief period of sustained work at moderate intensity and with inadequate sleep constitutes a potent multi-stressor condition capable of compromising whole-body exercise performance that requires physical and mental effort.

The individual contribution of each stressor, as well as the cumulative volume of stress encountered in the current SUSOPS cannot be determined directly, since no measurements were performed in the field (i.e., estimates of caloric deficit, nap architecture, or physical and mental strain). Yet, specific physiological and psychological modifications, typical of such multi-stressor tasks (Nindl et al. 2002; Rognum et al. 1986; Bahr et al. 1991; Lieberman et al. 2005, 2006), could be observed during the resting phase preceding the SUSOPS cycling trials. Namely, the reductions in body mass and RER, and the elevation in basal HR denoted an induction of a “stress response” to the SUSOPS stimuli. Such a reaction was further substantiated by the self-reported prevalence of negative affects (i.e., depression, anger, and confusion), excessive sleepiness, enhanced levels of perceived fatigue, and impaired intrinsic motivation.

SUSOPS did not affect the exercise-induced elevation in cardiac stroke volume and output. Towards the end of both CLT_85_ trials, however, SV declined, and there was a statistical tendency also for a CO drop; these reductions occurred at an earlier stage in the SUSOPS trial. The underlying mechanisms of this accelerated drop in SV are difficult to discern from current results, and remain hypothetical. We speculate that the accelerated SV fall was attributable to a decrease in ventricular end-diastolic volume (cardiac preload) (Gonzalez-Alonso and Calbet 2003; Gonzalez-Alonso et al. 2004), secondary to hypovolemia (Wittels et al. 1996), and hypohydration (Lieberman et al. 2005), which may occur transiently during the initial phases of prolonged military exercises. Although some evidence of transient myocardial dysfunction following ultra-endurance events exist (Douglas et al. 1987), no indications of excessive cardiac fatigue has been detected following a considerably longer (6 day) and more intense military training than the present SUSOPS (Opstad et al. 1994).

Notably, a blunted pressor response to exercise stimulus was observed during the SUSOPS trial. Based on previous evidence (Opstad 1990), the lower exercise pressor reaction was probably related wholly, or to a large extent, to peripheral adrenergic desensitisation elicited by sustained elevations of circulating norepinephrine during multi-stressor conditions. Due to technical limitations, we were not able to monitor MAP during the CLT_85_ trial. Considering the blunted pressor response in the SUSOPS CLT_65_ trial, however, it appears highly unlikely that enhanced ventricular afterload could have contributed to the accelerated terminal SV fall during the SUSOPS CLT_85_ trial (cf. Gonzalez-Alonso and Calbet 2003). Nevertheless, the present results clearly demonstrate that the stress during a short-term military operation may elicit cardiovascular instability, which in turn precipitates a reduction in exercise endurance.

SUSOPS increased submaximal $\dot {V}$O_2_ during cycling performed at fixed absolute workloads, indicating a reduction in whole-body mechanical efficiency (Bahr et al. 1991). Considering that the locomotive muscle aerobic metabolism, as determined by the NIRS measures on the vastus lateralis, was not perturbed by SUSOPS, the greater pulmonary $\dot {V}$O_2_ values were probably attributable to higher metabolic demands of other tissues; for instance, of the metabolic costs associated with the exaggerated exercise hyperpnoea and the hyperkinetic circulation in the SUSOPS trials. It is also plausible that, following SUSOPS, the amount of liver and skeletal muscle glycogen was relatively low, as suggested by the lower RER (i.e., enhanced rate of lipolysis) and capillary [La] (Smith et al. 2016; Rognum et al. 1981); a condition that may explain, at least partly (Bahr et al. 1991), the increase in submaximal values of systemic $\dot {V}$O_2_, as well as of HR, $\dot {V}$E and RPE (Lima-Silva et al. 2011; Carter et al. 2004; Heigenhauser et al. 1983).

Although leg tissue oxygenation did not vary at submaximal intensities, it is noteworthy that, despite the shorter duration of the SUSOPS trial, both CLT_85_ were terminated at similar degrees of muscle deoxygenation. This peripheral response, which coincided with the apparent CO drop at the exhaustive point, was presumably governed by central cardiovascular restraints, thus reflecting the inability of the heart to maintain O_2_ delivery to exercising muscles (cf. Gonzalez-Alonso and Calbet 2003). In addition, it cannot be ruled out that the attainment of a critical threshold of leg-muscle deoxygenation, reflecting a similar amount of peripheral fatigue, might have provided inhibitory somatosensory feedback on central motor drive, limiting exercise performance (see Amann 2011). In this regard, the observation that all subjects of the study regarded “intolerable leg pain” as the main determinant of the SUSOPS trial is of interest. Such a response, however, was not associated with excessive metabolic acidosis, which, in fact, appeared to be diminished by SUSOPS, judging by the lower values of capillary [La], and the lower peak values of $\dot {V}$E, $\dot {V}$CO_2,_ and RER. Interestingly, Marcora et al. (2008) have shown that, independently of metabolic stress, locomotor muscle fatigue may aggravate exercise-induced cardiorespiratory strain, possibly by way of increased central motor command.

SUSOPS did not influence the cerebral tissue oxygenation response to exercise. Yet the magnitude of cerebral deoxygenation incurred at the exhaustive point was attenuated by SUSOPS, a response that was probably associated with the brief exercise duration in this condition. Hence, it is reasonable to assume that, following SUSOPS, the voluntary termination of exercise was not driven by the oxygenation status of the prefrontal cortex; presumably, the hastened reduction in systemic O_2_ delivery, which coincided with the attainment of similar levels of leg-muscle deoxygenation (i.e., peripheral fatigue), precipitated task failure, prior to the development of cerebral hypoxia. However, considering that NIRS measurements reflect but region-specific oxygenation changes, we cannot exclude that other brain areas, critically involved in strenuous exercise (cf. Jung et al. 2015), were affected by SUSOPS.

In line with results from the previous military training studies (Lieberman et al. 2005, 2006), the 2-day SUSOPS degraded mood, and enhanced the perceived sensation of fatigue. The negative affective state prevailed throughout the constant-load trials; indeed, at the point of exhaustion, the decline in FS ratings was more pronounced in the SUSOPS trial, despite its shorter duration. At submaximal intensities, SUSOPS also compounded the exercise-induced elevations in RPE, presumably reflecting either increased afferent sensory feedback from different tissues (i.e., greater $\dot {V}$E, $\dot {V}$O_2,_ and HR; Hampson et al. 2001) and/or enhanced corollary discharge (de Morree et al. 2012). Still, at task failure, the final values of RPE did not differ between trials. Affect and effort perception constitute prime drivers of decision making and intrinsic motivation (Cabanac 1971), and non-pleasurable sensations may impose, consciously, and/or subconsciously, critical determinants to whole-body endurance performance (Marcora et al. 2008, 2009, St Clair Gibson et al. 2017, St Clair Gibson and Noakes 2004). Collectively, the present findings may imply, therefore, that the stress induced by short-term military operations provokes physiological, as well as psychological perturbations, which in an interactive manner, accelerate exercise-induced fatigue.

The self-reported feelings of depression and anger, which were aggravated by SUSOPS, were somewhat ameliorated immediately after the termination of the trials. An affective “rebound” from negativity to positivity has typically been observed following acute exercise; psychological (e.g., increased sense of self-accomplishment) and/or physiological (e.g., increased secretion of endorphins) mechanisms seem to underpin such exercise-induced mood enhancement (cf. Ekkekakis et al. 2011). In this regard, there is also evidence that light-to-moderate physical activity mitigates, partly and shortly, the adverse effects of sleep deprivation on affective state (Temesi et al. 2013; JrLeDuc et al. 2000). Still, the current finding showing that acute exhaustive exercise provokes a selective diminution of SUSOPS-engendered negative affects is of interest, and needs to be investigated further.

Due to the difficulties of blinding subjects to the SUSOPS intervention, and the lack of a control group (i.e., without SUSOPS), we cannot rule out that present results might have been influenced, at least to some extent, by a nocebo effect. In addition, we are not able to determine any gender differences, since only one female subject was investigated; her physiological and psychological reactions, however, conformed to those obtained by the majority of the subjects. Moreover, CO and SV were estimated noninvasively using electrical impedance cardiography, which has been validated previously during maximal exercise conditions (Siebenmann et al. 2015; Richard et al. 2001). Nevertheless, the study would have benefited from the employment of a direct method (i.e., Fick method). Finally, the study was designed a priori to examine the effects of SUSOPS on cycling performance at the same absolute work intensity. It remains uncertain whether the 2-day SUSOPS impaired subjects’ $\dot {V}$O_2peak_, thereby shifting the absolute workload to greater relative exercise intensity.

## Conclusion

Present findings indicate, in a group of well-trained individuals, that physiological and psychological perturbations evoked by short-term (2 days) military sustained operations with partial sleep deprivation accelerate the development of fatigue during whole-body strenuous endurance exercise.

## Abbreviations

CI: Confidence interval
CLT_65_: A 15-min constant-load trial at 65% of peak power output
CLT_85_: Exhaustive constant-load trial at 85% of peak power output
CO: Cardiac output
CON: Control trial
DAP: Diastolic arterial pressure
FAS: Perceived activation
*f*_R_: Respiratory frequency
FS: Affective valence
HR: Heart rate
[La]: Blood lactate concentration
MAP: Mean arterial pressure
MFI: Multidimensional Fatigue Inventory
NIRS: Near-infrared spectroscopy
*P*_ET_CO_2_: Partial pressure of end-tidal carbon dioxide
POMS-SF: Profile of Mood States-Short Form
PPO: Peak power output
RER: Respiratory exchange ratio
RPE: Ratings for perceived exertion
SAP: Systolic arterial pressure
SD: Standard deviation
SUSOPS: Sustained operations
SV: Stroke volume
TOI: Tissue oxygen index
$$\dot {V}$$E: Expired ventilation
$\dot {V}$E/$\dot {V}$CO_2_: Ventilatory equivalent for carbon dioxide
$\dot {V}$E/O_2_: Ventilatory equivalent for oxygen
$\dot {V}$CO_2_: Carbon dioxide production
$$\dot {V}$$O_2_: Oxygen uptake
$\dot {V}$O_2peak_: Peak oxygen uptake
*V*_T_: Tidal volume
Δ[HbO_2_]: Changes in oxyhaemoglobin
Δ[HbO_2_]: Changes in deoxyhaemoglobin
